# An all manganese-based oxide nanocrystal cathode and anode for high performance lithium-ion full cells†

**DOI:** 10.1039/c9na00003h

**Published:** 2019-03-11

**Authors:** Song Chen, Yumeng Shi, Ye Wang, Yang Shang, Wei Xia, Hui Ying Yang

**Affiliations:** International Collaborative Laboratory of 2D Materials for Optoelectronics Science and Technology of Ministry of Education, College of Optoelectronic Engineering, Shenzhen University Shenzhen 518060 China yumeng.shi@szu.edu.cn; Pillar of Engineering Product Development, Singapore University of Technology and Design 8 Somapah Road 487372 Singapore yanghuiying@sutd.edu.sg; Engineering Technology Research Center for 2D Material Information Function Devices and Systems of Guangdong Province, College of Optoelectronic Engineering, Shenzhen University Shenzhen 518060 China; Key Laboratory of Material Physics of Ministry of Education, School of Physics and Engineering, Zhengzhou University Zhengzhou 450052 China; College of Mechanical Engineering, Beijing University of Technology, Beijing Key Laboratory of Nonlinear Vibrations and Strength of Mechanical Structures Beijing 100124 China

## Abstract

Manganese oxide nanocrystals are of great interest for producing advanced high-performance lithium ion batteries owing to the shortened lithium ion diffusion length and accelerated interfacial charge transfer rate. Here we have developed a well-controlled generic method to synthesize monodisperse MnO nanocrystals, and present a comparative study regarding the effect of crystallite size on electrochemical stability. Nanocrystalline MnO with a size of about 10 nm shows the optimal lithium-storage performance. Notably, Mn-based nanocrystals retain their stable cyclability and excellent high-rate performance as both the anode and cathode. The all-nanocrystal MnO/C//LMO Li-ion full cells not only significantly improve the electrochemical properties of Mn-based materials but also open up avenues for the future development of various energy devices.

## Abstract 2

Manganese oxide nanocrystals are of great interest for producing advanced high-performance lithium ion batteries.

## Graphical abstract

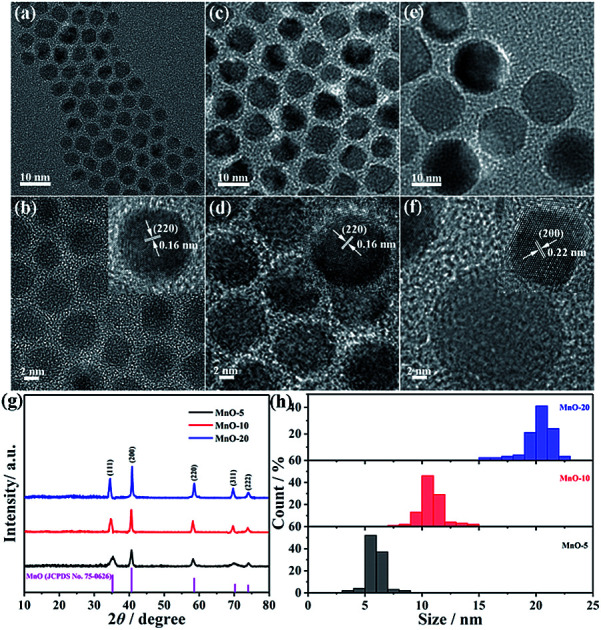

## Introduction

Designing monodisperse nanocrystals is one of the central topics in the fields of both fundamental research and technological applications due to their remarkable physical and chemical properties.^1–3^ In the past decade, a wide range of zero-dimensional nanomaterials, including metals, alloys, transition metal oxides, transition metal dichalcogenides, *etc.*, have been intensively investigated for use in catalysis, water treatment, sensors, and energy storage and conversion.^4–9^ Rationally designed ultra-small nanocrystals for lithium ion batteries (LIBs) can significantly shorten the lithium ion diffusion pathway, facilitate interfacial charge transfer and enlarge the electrode/electrolyte interface, thus improving electrochemical reaction kinetics.^10–16^

Manganese oxide (MnO) has attracted great interest as a promising candidate for the next-generation LIBs because of its high theoretical capacity (755 mA h g^−1^), relatively low electromotive force (1.032 V *vs.* Li^+^/Li), high abundance, low cost and environmental benignity.^17,18^ However, the use of MnO is still hindered by inferior rate capability caused by kinetics limitations and poor cycling stability resulting from severe volume change during repeated cycles.^19–22^ MnO nanocrystals (MnO NCs) have a large surface area and shorter Li ion diffusion distance, which provide more active sites and specific facets for improved electrochemical properties.^23,24^

Based on electrochemical measurements and theoretical analyses, Okubo *et al.* have investigated the effect of nanosize on a nanocrystalline LiCoO_2_ electrode and revealed that extremely small crystallite size was unfavorable for energy storage applications.^25^ It was reported that the crystallite size of spinel LiMn_2_O_4_ below 15 nm provides good cyclability at a high rate due to the lithiation without domain boundaries.^26^ Qian *et al.* have demonstrated a change of the electron spin state associated with lithium insertion/extraction in nanosized LiCoO_2_, implying that it is promising to alter the electronic properties different from the bulk behavior by adjusting the surface of nanomaterials.^27^ The lithium-storage mechanism of MnO based on the reversible conversion reaction between Li and MnO can be enabled by the generation of nanosized Mn grains dispersed in the Li_2_O matrix. In particular, the nanosized grains can facilitate kinetically the reversible reaction by creating a large contact surface.^28^ Zhong *et al.* have reported that MnO particles composed of MnO grains within the nanosize range could be lithiated at a higher potential (0.4 V) than bulk MnO.^29^ However, nanocrystalline MnO with extremely small size can undergo severe agglomeration during phase transformation of the reverse reaction, deteriorating the lithium-storage performance.^30^ At this point, size-controllable synthesis of MnO anode materials may be worth exploring to realize a superior performance. To date, although tremendous well-designed MnO nanoarchitectures have been constructed to tackle the above issues of cycling and rate properties, there are only a few reports on the synthesis of monodisperse size-controlled MnO nanocrystals for LIB anodes and the comparative study for electrochemical performance.

In addition, most of the existing related studies mainly focus on using nanomaterials with small size for anodes, while the cathode materials are mostly based on the conventional large-sized microspheres or nanoparticles of LiCoO_2_ and LiFePO_4_.^31–33^ Recently, it has been recognized that LiMn_2_O_4_, LiMnO_2_, and the emerging Li-rich Mn-based materials are desirable substitutes for traditional LiCoO_2_ cathodes.^12^ However, so far, the simultaneous realization of a nanocrystal anode and a nanocrystal cathode for full cell assembly has been rarely reported.

In this work, we propose a new method to synthesize monodisperse MnO nanocrystals with uniform and controllable sizes. As a lithium ion battery anode, nanocrystalline MnO with a crystallite size of about 10 nm shows the optimal electrochemical stability. The MnO anode is also designed to form nanocomposites with carbon materials, which can serve as the conductive network and buffer layer to improve electrical conductivity and alleviate structural stress during cycling. The MnO/C nanocomposite anodes realize significantly enhanced lithium-storage properties (high reversible capacity of 806.7 mA h g^−1^ at 200 mA g^−1^ after 150 cycles), excellent cycling stability (capacity retention of 92.1% after 150 cycles) and good rate capability (466.6 mA h g^−1^ at 2 A g^−1^). Furthermore, spinel LiMn_2_O_4_ nanocrystals (LMO NCs) with a relatively homogeneous size and morphology have been obtained after the chemical lithiation of MnO NCs *via* a simple solid-state reaction. As a lithium ion battery cathode, nanocrystalline LiMn_2_O_4_ also exhibits a high capacity retention of 91.8% at 1C after 100 cycles, and an outstanding rate capability of 84.6 mA h g^−1^ at a high rate of 10C. An all-nanocrystal MnO/C//LMO Li-ion full cell was assembled as a commercialization effort and it shows good lithium-storage performance. Therefore, our findings may shed some light on how to rationally design unique nanoarchitectured materials for various energy devices.

## Results and discussion

**Fig. 1:**
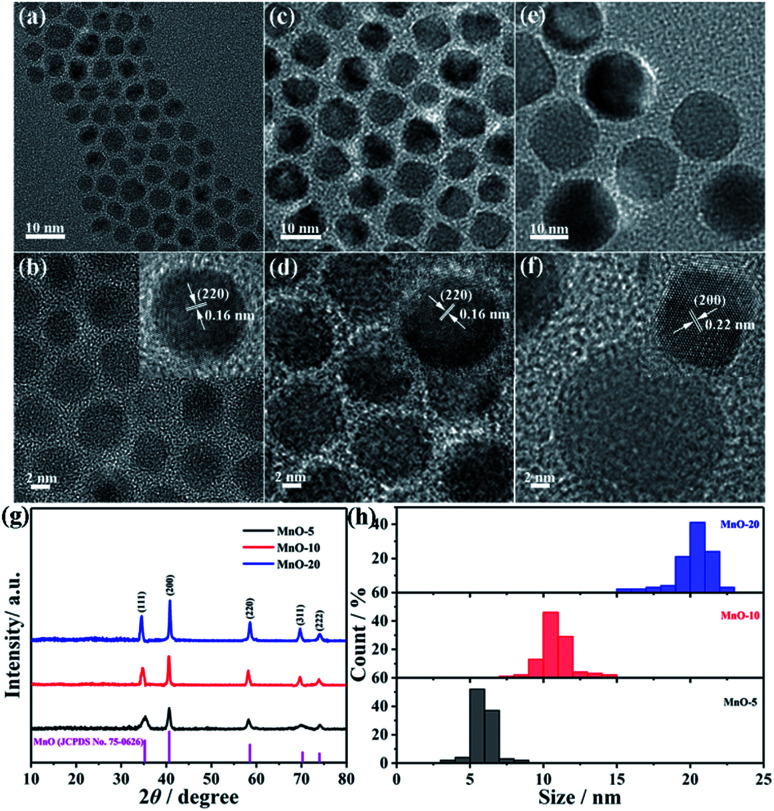
TEM and HRTEM images of (a and b) MnO-5, (c and d) MnO-10 and (e and f) MnO-20 NCs. (g) XRD patterns and (h) size distributions of MnO NCs.

In this work, MnO NCs with different sizes were synthesized. The crystal size was controlled effectively by varying reaction time, reaction temperature and solvents. As observed from the TEM images, nearly monodisperse MnO NCs were obtained with average diameters of around 5 nm, 10 nm and 20 nm, respectively (Fig. 1a–f). And for convenience, MnO NCs are denoted as MnO-5, MnO-10 and MnO-20, respectively. The HRTEM images show clear lattice fringes, indicating the high crystallinity of MnO NCs (Fig. 1b, d and f). The lattice spacings of 0.16 nm and 0.22 nm match well with the (220) and (200) planes of cubic MnO. Fig. 1g shows the size-dependent XRD patterns of nanocrystals. All the diffraction peaks correspond to the MnO cubic structure (JCPDS card no. 75-0626), implying the formation of a pure phase with good crystallinity without any impurity. The grain size of samples was estimated using the Scherrer formula based on (111) peaks. The calculated sizes (*L*) are about 5.3, 11.1 and 22.5 nm, respectively, which are approximately consistent with TEM results. In addition, the size distribution of nanocrystals is shown in Fig. 1h, further confirming the uniform diameter distribution and size-controlled synthesis of MnO NCs. After counting about 100 nanocrystals, the mean sizes are calculated to be 5.3 ± 1.1, 10.4 ± 1.7 and 20.5 ± 2.1 nm, respectively. According to the Lifshitz–Slyozov–Wagner (LSW) model and Gibbs–Thomson equation,^34–36^ particle growth is driven by the diffusion of solute molecules, from small to large size, and the following relationship can be obtained:1
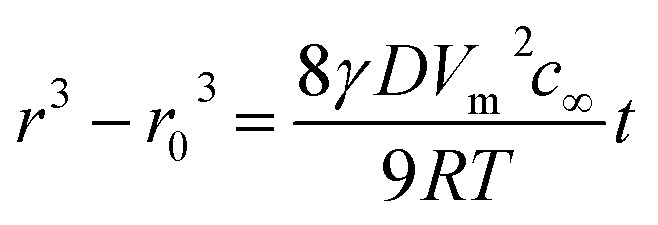
where *r* and *r*_0_ are the particle radius at time *t* and 0, respectively, *R* is the gas constant, *T* is the absolute temperature, *γ* is the surface energy of the solid, *D* is the diffusion coefficient of the solute, *V*_m_ is the molar volume, and *c*_∞_ is the solubility at a flat surface. From eqn (1), the increase of crystal size has a rough linear correlation with reaction time at a fixed temperature. Therefore, with the increase of reaction time from 30 min to 6 h, the size of nanocrystals increased from ∼10 nm to ∼20 nm when ODE was used as the solvent. Moreover, narrow size distributions can be realized in solvents with low boiling point.^37^

**Fig. 2:**
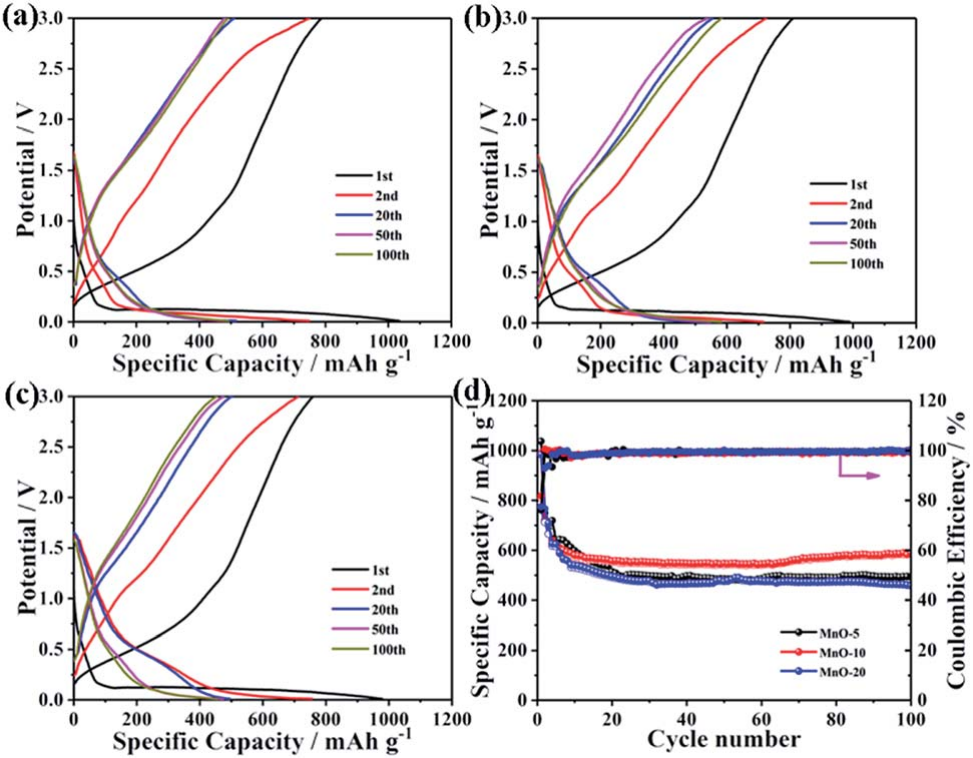
Galvanostatic charge–discharge profiles of (a) MnO-5, (b) MnO-10 and (c) MnO-20 NCs at 100 mA g^−1^. (d) Charge and discharge capacities of three electrodes at a current density of 100 mA g^−1^.

In order to verify the influence of crystallite size, the electrochemical performance of MnO NC anodes with different sizes was evaluated. The galvanostatic charge–discharge curves of three electrodes for the 1st, 2nd, 20th, 50th and 100th cycles at 100 mA g^−1^ are shown in Fig. 2a–c. The MnO-10 NC anode delivers first discharge and charge capacities of 989.3 mA h g^−1^ and 809.2 mA h g^−1^, respectively, with an initial coulombic efficiency of 81.8% (Fig. 2b). However, the MnO-5 and MnO-20 NC electrodes deliver capacities of 1036.6/789.7 mA h g^−1^ and 984.2/762.1 mA h g^−1^, respectively (Fig. 2a and c), with initial coulombic efficiencies of 76.2% and 77.4%, respectively. MnO-10 NCs show the highest reversible specific capacity, and from the cycling performance curves, it can be clearly observed that the cycling stability increased with a coulombic efficiency of approximately 100% from the second cycle onward (Fig. 2d). The corresponding capacity retention is about 72.2% against the second cycle, and only 62.4% and 60.3% for the MnO-5 and MnO-20 NCs, respectively. From the above results, the as-synthesized MnO-10 NCs show the best electrochemical lithium-storage performance. Generally speaking, the decrease of crystallite size could significantly shorten the lithium ion diffusion length and provide sufficient electrochemically active sites and lithium insertion/extraction channels, thus providing outstanding long-term cycling stability.^38^ However, excessive surface atoms and specific facets exposed to the outside caused by the extremely small size could lead to more side reactions with the electrolyte, resulting in the deterioration of cycling stability.^26^ In addition, based on the lattice gas model and assuming that the lithium site occupancy obeys the Fermi distribution,^25,39^ the decrease of crystallite size can give rise to more surface layers and induce more severe capacitor behavior. Therefore, nanocrystalline MnO with exceedingly small size is unfavorable for lithium ion battery anode materials.

**Fig. 3:**
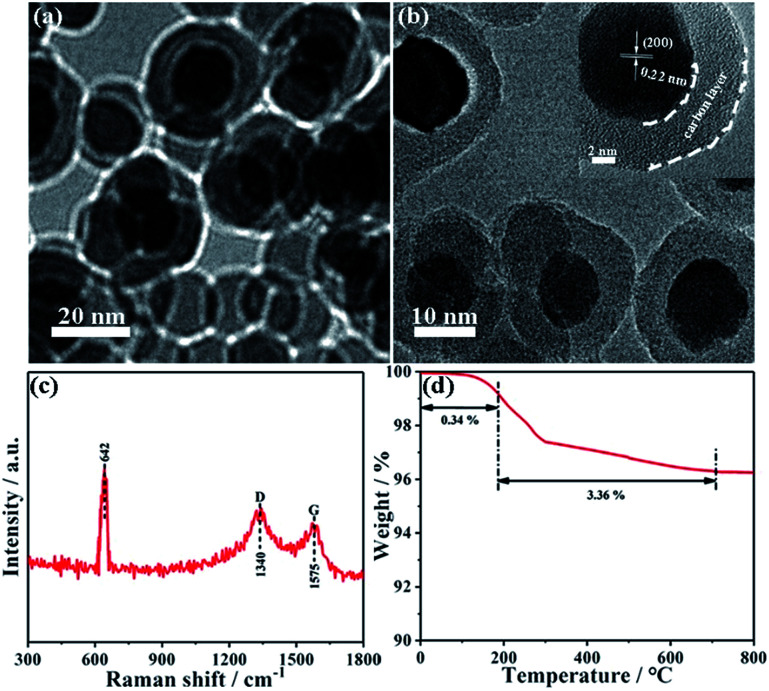
(a) TEM and (b) HRTEM images of MnO-10/C NCs. (c) Raman spectrum of MnO-10/C NCs. (d) TGA-DSC curves of MnO-10/C NCs.

Besides designing ultrasmall MnO nanoarchitectures, carbon decoration also provides an advanced method to further enhance the electrochemical lithium storage performance. Therefore, the MnO-10/C composite was prepared by coating the PS polymer with a simple chemical bath process followed by the carbonization of PS. As shown in Fig. S1,† with the exception of few multi-core/shell nanocomposites, one or two MnO nanocrystals are uniformly encapsulated by PS with an average shell thickness of 8 nm. The TEM image clearly shows that the as-prepared MnO-10/C nanocomposites almost retain the original morphology with the size range from 20 to 30 nm (Fig. 3a). As seen from the HRTEM image, MnO nanocrystals are roughly encapsulated by an amorphous carbon shell with ∼5 nm thickness, implying a slight shrinkage after carbonization at high temperature (Fig. 3b). The results indicate that the MnO cores haven't grown obviously, in spite of the calcination at high temperature in the process of carbonization. The increase of individual nanocrystal size may be due to the agglomeration and growth of two or more cores during the calcination process. In addition, the lattice spacing of 0.22 nm could be readily indexed to MnO (200), further confirming that MnO maintains high crystallinity. The XRD pattern of the MnO-10/C nanocomposite exhibits obvious diffraction peaks of cubic MnO, revealing that MnO crystals were well maintained during the carbonization process (Fig. S2†). The Raman spectrum of the nanocomposite material is shown in Fig. 3c. An obvious peak at around 642 cm^−1^ is related to Mn–O vibration mode.^40^ Two broad peaks observed at 1340 and 1575 cm^−1^ correspond to the D and G bands of carbon, respectively.^41^ TGA was performed in air to evaluate the carbon content (Fig. 3d). A weight loss below 200 °C could be ascribed to water dissipation. Subsequently, the continuous weight loss of the second stage until 700 °C is due to the combustion of carbon accompanied by the oxidation of MnO. According to previous reports,^40,42^ the oxidation process of MnO to Mn_2_O_3_ can lead to about 11.28% weight increase. From the above analysis, the actual carbon content is 3.36% weight loss plus 11.28% weight increase caused by the oxidation of MnO. Therefore, the carbon content in the MnO-10/C nanocomposite is about 14.6%. We investigated the Brunauer–Emmett–Teller (BET) specific surface area and porosity of MnO-10/C by nitrogen adsorption–desorption analysis (Fig. S3†). The isotherm is a type IV isotherm with a type H3 hysteresis loop, indicating a typical mesoporous structure. The MnO-10/C nanocomposites exhibit a BET specific surface area of 73.1 m^2^ g^−1^, which is higher than that of many previously reported MnO-based materials (Table S1†). Such a structure can not only favorably alleviate the volume change but also provide a continuous conductive network for the fast electron and Li^+^ transport.

**Fig. 4:**
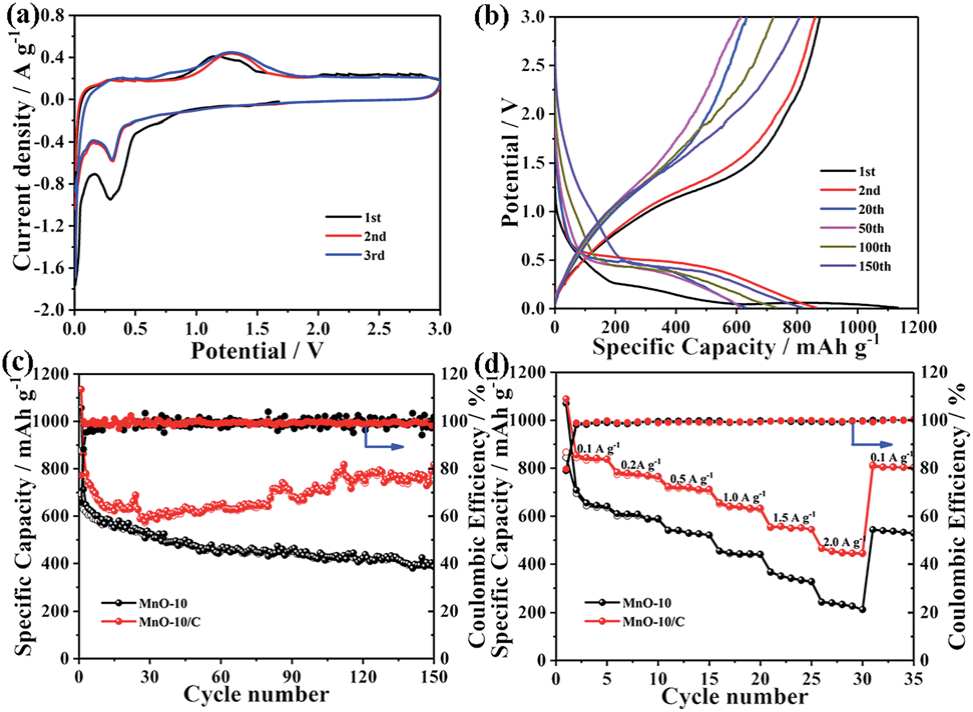
(a) CV curves of the MnO-10/C NC anode for the first three cycles. (b) Galvanostatic charge–discharge profiles of MnO-10/C NCs at 200 mA g^−1^. (c) Charge and discharge capacities of two electrodes at a current density of 200 mA g^−1^. (d) Rate capability of two electrodes at different current densities.

Fig. 4a shows the typical CV curves of the MnO-10/C NC electrode for the first three cycles. In the initial cycle, the cathodic peak located at about 0.75 V corresponds to the formation of solid electrolyte interface (SEI) films.^43^ This peak disappears in the subsequent cycles, indicating irreversible capacity loss. The other reduction peak at around 0.26 V is attributed to the reduction of Mn^2+^ to Mn^0^, which shifts to about 0.31 V from the second cycle onward.^44^ During the delithiation process, only one broad oxidation peak observed at around 1.21 V can be assigned to the oxidation of Mn^0^ to Mn^2+^, which shifts to around 1.26 V in the subsequent cycles. The above shifts of reduction and oxidation peaks may be due to the enhanced kinetics and material utilization of the MnO/C electrode caused by the nanostructure change after the first lithiation process.^45^ In addition, the CV curves of MnO/C nanocrystals basically overlap in the second and third cycles, revealing excellent electrochemical reversibility during the repeated charge/discharge processes.

The galvanostatic charge–discharge curves of MnO-10 and MnO-10/C electrodes for the 1st, 2nd, 20th, 50th, 100th and 150th cycles at 200 mA g^−1^ are shown in Fig. 4b and S4.† The first discharge and charge capacities of the MnO/C composite electrode are 1135.4 and 876 mA h g^−1^, respectively, with an initial coulombic efficiency of 77.2% (Fig. 4b). The large irreversible capacity loss is mainly attributed to the unavoidable formation of the SEI layer on the electrode surface, which is consistent with the above CV analysis. However, from the second cycle onward, the MnO/C composite anode exhibits outstanding cycling stability. By contrast, the MnO NCs only deliver initial discharge and charge capacities of 1056.7 and 694.8 mA h g^−1^, respectively, with a low initial coulombic efficiency of 65.7% (Fig. S4†). Compared with the pure MnO NCs, the first coulombic efficiency of the MnO/C nanocomposite is effectively improved.

To further investigate the significant influence of the carbon layer on cycling performance, the capacity difference of MnO-10 and MnO-10/C nanocrystals is shown in Fig. 4c. It could be clearly observed that pure MnO NCs exhibit severe capacity fading and unstable coulombic efficiency, which may result from severe particle agglomeration and volume change during repeated cycles. But surprisingly, after the MnO nanocrystals were decorated with a carbon layer, the cycling stability is significantly improved. The corresponding coulombic efficiency increases to over 95% in the 2nd cycle and is close to 100% in the subsequent cycles. After 150 cycles, the MnO/C composite electrode shows a stable reversible specific capacity of 806.7 mA h g^−1^ with a capacity retention of 92.1%. However, the specific capacity of MnO-10 NCs is 393.3 mA h g^−1^ after cycling, with a low capacity retention of 56.6%. Furthermore, it is also noteworthy that the specific capacity of the MnO/C nanocomposite decreases first and then increases during cycling. This U-shaped curve for cyclability has been observed for other MnO-based anodes reported previously, which may be ascribed to the generation of products with higher oxidation states, mixing effects of Mn cluster aggregation and enhancement of conversion reaction kinetics in MnO caused by slight defect formation.^46–49^ To further highlight the electrochemical performance of the MnO-10/C nanocomposite, we have compared various MnO-based anode materials reported previously (Table S2†). Remarkably, the MnO/C nanocomposite in our work shows high reversible specific capacity and outstanding cycling stability.

Fig. 4d shows the rate capability of MnO-10 and MnO-10/C nanocrystals. The MnO/C nanocomposite electrode delivers reversible discharge capacities of 1089.0, 783.2, 723.6, 656.5, 554.7 and 466.6 mA h g^−1^ at current densities of 0.1, 0.2, 0.5, 1.0, 1.5 and 2.0 A g^−1^, respectively. When the current density drops to 0.1 A g^−1^, a specific capacity of 812.8 mA h g^−1^ can still be recovered. In comparison, the pure MnO NCs under identical testing conditions exhibit low reversible specific capacities of 1070.0, 610.4, 541.7, 455.1, 367.7, 244.6 and 543.7 mA h g^−1^, respectively. Obviously, the MnO/C nanocomposite shows higher reversible specific capacity, more excellent cycling stability and better rate capability, which can be ascribed to the carbon layer which enhances the electrical conductivity, as demonstrated by the EIS testing (Fig. S5†). Moreover, such a nanostructure significantly inhibits the agglomeration of manganese grains and facilitates the conversion reaction during the repeated lithiation/delithiation process.^29,30,47^

**Fig. 5:**
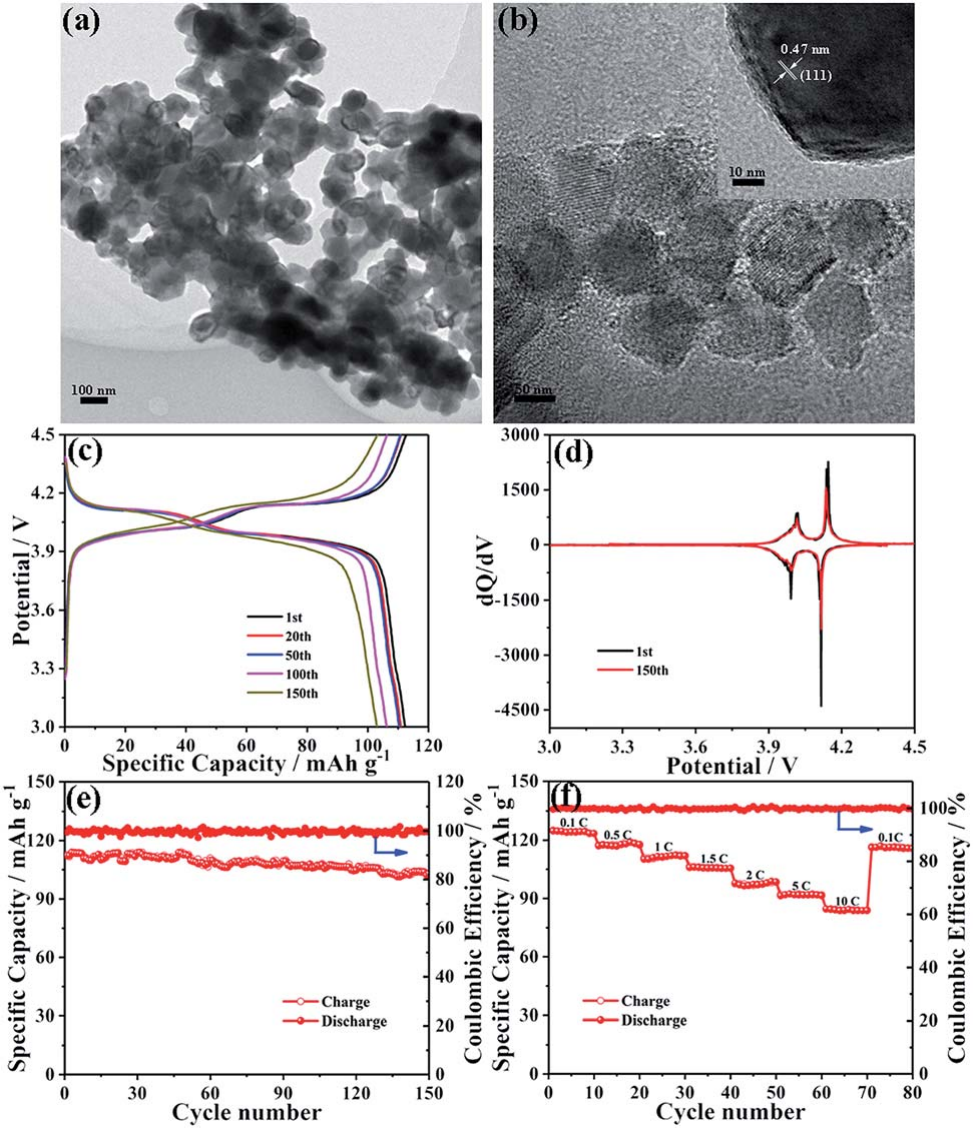
(a) TEM and (b) HRTEM images of LMO NCs. (c) Galvanostatic charge–discharge profiles and (d) the corresponding d*Q*/d*V* plots of LMO NCs at 1C. (e) Charge and discharge capacities of LMO NCs at 1C. (f) Rate capability at different current densities.

LiMn_2_O_4_ nanocrystals were synthesized by a solid state reaction of MnO nanocrystals and a Li–oleate complex. As seen from the TEM image shown in Fig. 5a, the obtained LMO NCs have a nearly well-dispersed morphology with an average diameter of ∼100 nm. Compared with MnO-10 NCs, the expansion of crystallite size may be due to the solid-state reaction at high temperature. The characteristic lattice fringe corresponding to 0.47 nm could be indexed to the (111) plane of spinel LiMn_2_O_4_ (Fig. 5b). The XRD pattern of LMO NCs displays the typical feature of the spinel structure with the *Fd*3*m* space group (JCPDS card no. 89-0106), without any peaks of impurity (Fig. S6†). The BET specific surface area of LiMn_2_O_4_ nanocrystals is measured to be about 45.7 m^2^ g^−1^, and the most probable pore diameter is about 2.3 nm (Fig. S7†). To evaluate the electrochemical performance of LMO NCs as cathode materials, a coin-type cell was also assembled. The galvanostatic charge–discharge curves of the LMO electrode for the 1st, 20th, 50th, 100th and 150th cycles at 1C (1C = 148 mA g^−1^) are shown in Fig. 5c. The charge and discharge curves both show two-step flat plateaus at about 3.9 and 4.1 V, suggesting the typical electrochemical behavior of LiMn_2_O_4_. The initial charge and discharge capacities are 112.7 and 112.2 mA h g^−1^, respectively, with a high coulombic efficiency of over 99%. The corresponding voltage *versus* d*Q*/d*V* profiles for 1st and 150th cycles are illustrated in Fig. 5d. As shown, both charge and discharge processes exhibit two pairs of distinct sharp peaks based on two different insertion or extraction reactions,^50–53^ which is in accordance with the above result shown in Fig. 5c. Additionally, the two curves after the 1st and 150th cycles are almost identical, implying the excellent electrochemical reversibility of the LMO NC electrode. The cycling performance of LMO NCs is shown in Fig. 5e. After 150 cycles, a specific capacity of around 103.0 mA h g^−1^ can still be maintained with an excellent capacity retention of 91.8%. Fig. 5f exhibits the rate capability of the LMO cathode at various charge/discharge rates. The discharge capacities of spinel LMO NCs at 0.1, 0.5, 1, 1.5, 2, 5 and 10C are 124.7, 117, 110.2, 105.9, 97.9, 91.4 and 84.6 mA h g^−1^, respectively. Moreover, when the current rate returns to 0.1C, a reversible capacity of 116.1 mA h g^−1^ can be recovered. Such an excellent rate performance can be mainly attributed to the low-dimensional nanoarchitecture of cathode materials, which can reduce effectively the lithium ion diffusion length and accelerate significantly charge transfer during cycling even at a high rate.

**Fig. 6:**
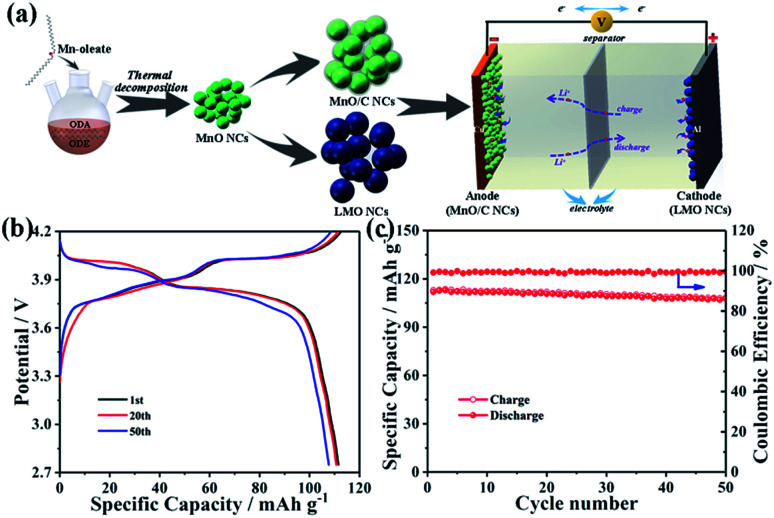
Electrochemical characterization of full cells. (a) Schematic of the synthesis procedure and full-cell configuration for Li-ion batteries with the MnO/C anode and LMO cathode. (b) Galvanostatic charge–discharge profiles and (c) cycling performance at 74 mA g^−1^ of the MnO/C//LMO full cell.

Finally, a prototype full cell composed of the MnO-10/C nanocomposite anode and the LMO nanocrystal cathode was assembled to further investigate its commercial viability (Fig. 6a). The specific capacity of the anode exceeds that of the cathode by ∼10%, which is calculated according to the weight of the LMO cathode. The full cell was tested at a current density of 74 mA g^−1^ between 2.75 and 4.2 V. As shown in Fig. 6b, the MnO/C//LMO full cell delivers reversible discharge capacities of 112.9, 111.9, and 108.2 mA h g^−1^ at the 1st, 20th, and 50th cycles, respectively. After 50 charge/discharge cycles, the capacity retention of the full cell is still over 90% with a coulombic efficiency of ∼100% for all cycles (Fig. 6c). In addition, the cycling performance of the MnO-10/C nanocomposite anode in the full cell was also investigated, where the specific capacity is calculated according to the weight of the MnO-10/C anode (Fig. S8†). The anode was prelithiated using lithium metal in a half-cell to form the stable SEI films prior to capacity matching with the cathode.^54,55^ As shown, the MnO/C//LMO full cell delivers reversible specific capacities of 801.2, 781.9, and 725.3 mA h g^−1^ at the 1st, 20th, and 50th cycles, respectively. After 50 charge/discharge cycles, the capacity retention is about 91%. The good full-cell stability further demonstrates the synergistic effects of the 0D nanocrystals and carbon layer on the electrode architecture. Therefore, this triumphant MnO/C anode and LMO cathode have clear commercial feasibility for next-generation battery systems.

## Conclusions

In summary, monodisperse and size-controlled MnO nanocrystals have been synthesized by a facile one-pot pyrolysis approach as a LIB anode. MnO NCs with a crystallite size of about 10 nm exhibit the best electrochemical stability. After decorating with a homogeneous carbon layer, the MnO/C nanocomposite electrode shows enhanced electrochemical properties, and delivers not only a stable reversible capacity of 806.7 mA h g^−1^ and a high capacity retention of 92.1% at 200 mA g^−1^ after 150 cycles, but also a good rate capability of 466.6 mA h g^−1^ at 2 A g^−1^. The improved lithium-storage performance can mainly benefit from the synergistic effect of the nanostructure and carbon decoration: (1) nanocrystals with suitable size could effectively shorten the lithium ion diffusion length, accelerate charge transfer and provide sufficient lithium-storage active sites; (2) the surface carbon layers are able to improve electrical conductivity, alleviate volume changes and suppress the agglomeration of manganese grains during the repeated lithiation/delithiation process. Moreover, the as-synthesized MnO NCs were chemically converted to LMO NCs through a simple solid-state method as a LIB cathode, which also displays high reversible Li-storage capacity with excellent cycling stability and high-rate performance. More significantly, the all-nanocrystal MnO/C//LMO Li-ion full cell is a promising candidate for the next generation of LIBs. These promising findings open up new avenues to construct unique nanostructures for future energy storage and conversion technologies.

## Conflicts of interest

There are no conflicts to declare.

## Supplementary Material

NA-001-C9NA00003H-s001
