# Supplementary material for: An all manganese-based oxide nanocrystal cathode and anode for high performance lithium-ion full cells
Source: Nanoscale Adv. 2019 Mar 11;1(5):1714–20. doi: 10.1039/c9na00003h (PMC9417273; doi:10.1039/c9na00003h)
Supplement: NA-001-C9NA00003H-s001 [file NA-001-C9NA00003H-s001.pdf]

Electronic Supplementary Material (ESI) for Nanoscale Advances.
This journal is © The Royal Society of Chemistry 2019

Supporting Information for

All Manganese-based Oxide Nanocrystal Cathode and Anode for High Performance Lithium-Ion Full-Cell

Song Chen,[a,b] Yumeng Shi,*[a,c] Ye Wang,[d] Yang Shang,[b] Wei Xia,[e] Hui Ying Yang*[b]

a. International Collaborative Laboratory of 2D Materials for Optoelectronics Science and Technology of Ministry of Education, College of Optoelectronic Engineering, Shenzhen University, Shenzhen 518060, China.

b. Pillar of Engineering Product Development, Singapore University of Technology and Design 8 Somapah Road, 487372, Singapore

c. Engineering Technology Research Center for 2D Material Information Function Devices and Systems of Guangdong Province, College of Optoelectronic Engineering, Shenzhen University, Shenzhen 518060, China

d. Key Laboratory of Material Physics of Ministry of Education, School of Physics and Engineering, Zhengzhou University, Zhengzhou 450052, China

e. College of Mechanical Engineering, Beijing University of Technology, Beijing Key Laboratory of Nonlinear Vibrations and Strength of Mechanical Structures, Beijing 100124, China

1. Experimental Details

Material preparation

Synthesis of Mn-oleate and Li-oleate complex. Mn-oleate complex was synthesized by the procedure reported previously with some modifications.[1] In brief, 20 mmol manganese chloride tetrahydrate and 60 mmol sodium oleate were dissolved in the mixture of 30 ml distilled water, 40 ml ethanol and 70 ml hexane. Subsequently, the above solution was heated at 70 °C for 4 hours. After cooled to room temperature, the resulting upper organic layer containing Mn-oleate complex was washed several times with water and ethanol in the separatory funnel. Finally, Mn-oleate complex in a solid form was obtained after hexane was evaporated. Li-oleate complex was prepared by the reaction of lithium chloride and oleic acid. Typically, 20 mmol lithium chloride was dissolved in 20 mmol oleic acid. The mixture was subsequently heated to 200 °C and then kept for 24 hours. After cooled to room temperature, Li-oleate complex in a waxy solid form was obtained.

Synthesis of MnO NCs. In a typical process, 0.5 mmol Mn-oleate precursor, 2 mmol 1-octadecanol (ODA) and 20 mmol 1-octadecene (ODE) were added in the three-necked flask. The above mixture was degassed at 120 °C for 1 hours under argon atmosphere, and then heated to 310 °C and kept for 30 mins. After cooled to room temperature, MnO NCs could be obtained by centrifuging several times with ethanol to remove excess surfactant and solvent.

The size of MnO NCs can be controlled effectively by adjusting synthesis parameters (reaction time, reaction temperature or solvent). When the reaction time increased from 30 mins to 6 hours, the size of nanocrystals increased from ~10 nm to ~20 nm. When 1-hexadecene (HDE) was used as solvent to displace ODE and the reaction was performed at 280 °C for 30 mins, nanocrystals of ~ 5 nm were obtained. For convenience, MnO NCs of about 5 nm, 10 nm and 20 nm are denoted as MnO-5, MnO-10 and MnO-20, respectively.

Synthesis of MnO/C NCs. The as-synthesized MnO NCs were dispersed in dimethylformamide (DMF), and then polystyrene (PS) was added in above solution.

Ultimately, MnO/C composite NCs were obtained by calcining at 600 ℃ for 5 hours under Ar with a heating rate of 2 ℃ min^{-1}.

Synthesis of LMO NCs. The as-synthesized MnO NCs were mixed homogeneously with the stoichiometric amounts of Li-oleate. LMO NCs were ultimately obtained by calcining at 500 ℃ for 5 hours in air with a heating rate of 2 ℃ min^{-1}.

Material characterization

X-ray diffraction (XRD) patterns of all samples were performed using PANalytical X-pert diffractometer (PANalytical, Netherlands) with Cu Kα radiation source operated at 40 kV and 40 mA. The morphology and structure were observed using transmission electron microscopy (TEM, JSM-2100F), and high-resolution transmission electron microscopy (HRTEM, FEI Tecnai G2 F20). Content measurements were carried out using thermogravimetric analysis (TGA, Shimadzu DTG-60) in air.

Electrochemical Measurements

Electrochemical properties of nanocrystals were investigated by a typical half-cell configuration with CR2025 type coin cells. To prepare working electrodes, active materials (MnO NCs, MnO/C NCs and LMO NCs), acetylene black and polyvinylidene fluoride (PVDF) at a weight ratio of 8:1:1 were dispersed in 1-methyl-2-pyrrolidinone (NMP) to form a uniform slurry. The slurry was coated on the copper/aluminum foil, and then vacuum dried at 100℃ overnight. The separators and electrolyte were Celgard 2400 membranes and 1M $LiPF_6$ dissolved into the mixture solvent of ethylene carbonate (EC) and dimethyl carbonate (DMC) (1:1 in the volume ratio), respectively. The galvanostatic charge/discharge tests of anodes and cathodes were performed using a LAND CT-2001A cell testing system at different current densities between 0 to 3.0 V and 3.0 to 4.5 V (vs. Li^+/Li), respectively. Cyclic voltammetry (CV) of anodes and cathodes was conducted using the IM6e electrochemical workstation at different scan rates in the potential range of 0-3.0 V and 3.0-4.5 V, respectively. Electrochemical impedance spectroscopy (EIS) was carried out on the same equipment in the frequency range from 100 kHz to 0.01 Hz.

The anode and cathode are MnO/C NCs and LMO NCs, respectively. The assembly process of full-cell was identical to that of the above half-cell. The galvanostatic charge/discharge measurements of MnO/C//LMO full cell was carried out at 74 mA g^{-1} from 2.75 to 4.2 V. The specific capacity was calculated according to the weight of LMO cathode.

2. Supplementary Figures

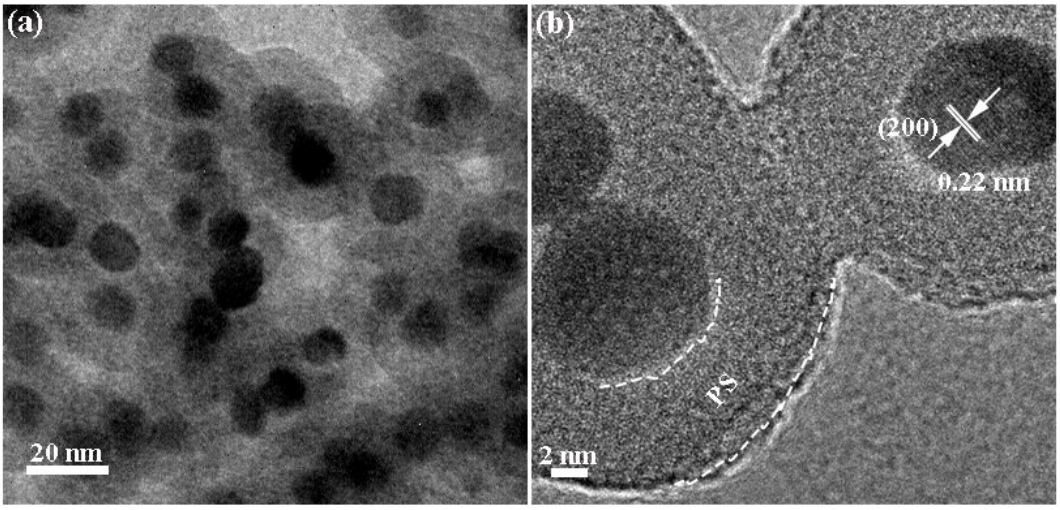

Fig. S1. TEM and HRTEM images of MnO/PS precursors.

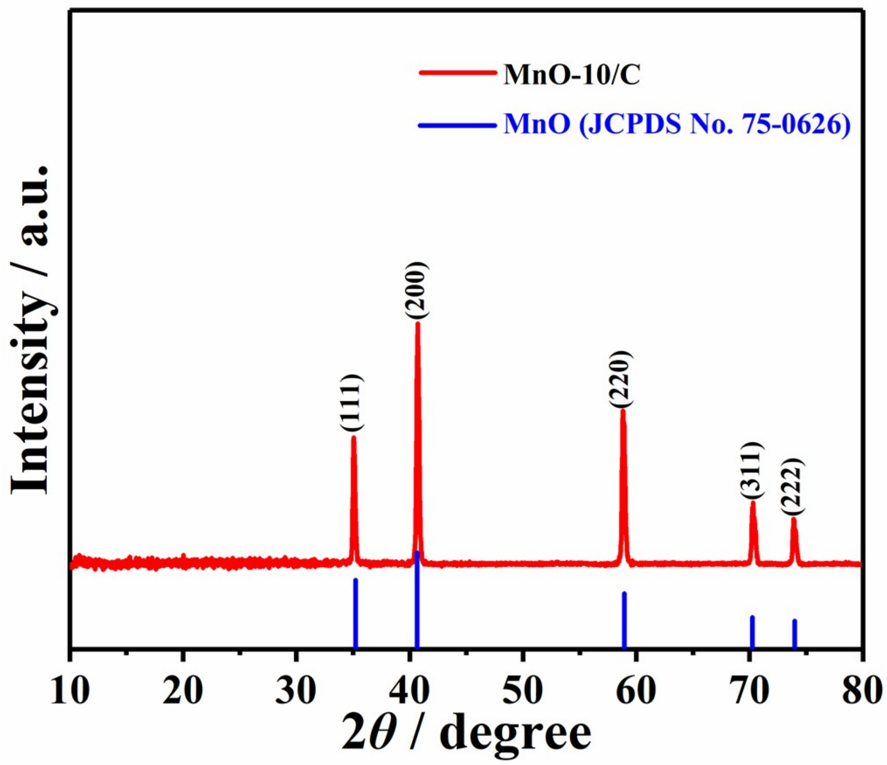

Fig. S2. XRD pattern of MnO-10/C NCs.

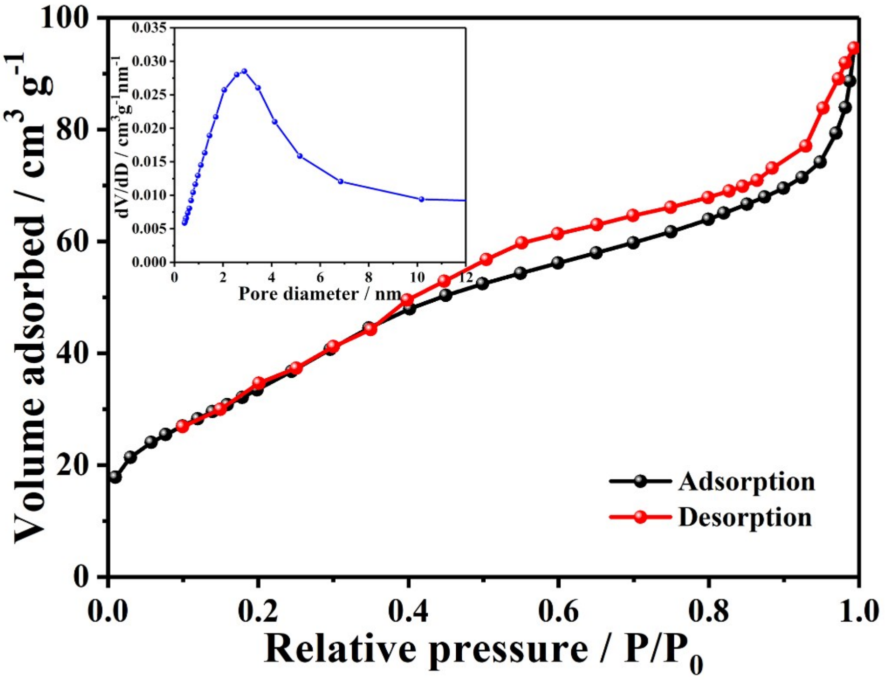

Fig. S3. N_2 adsorption/desorption isotherm curve and the corresponding pore size distributions of MnO-10/C nanocomposites.

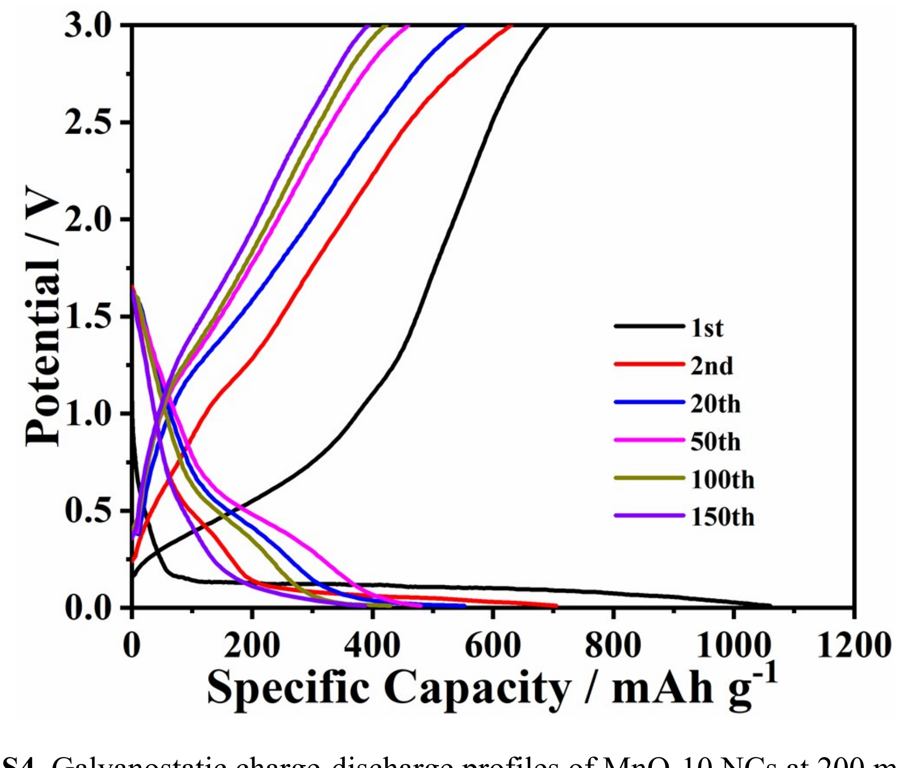

Fig. S4. Galvanostatic charge-discharge profiles of MnO-10 NCs at 200 mA g^{-1}.

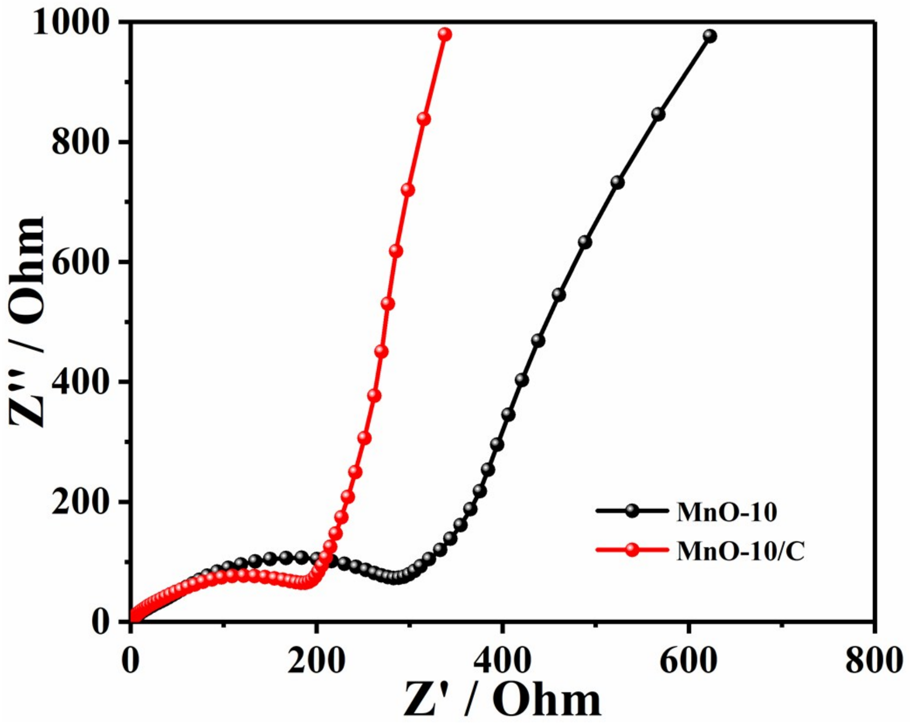

Fig. S5. Nyquist plots of MnO-10 and MnO-10/C NC electrodes before cycling.

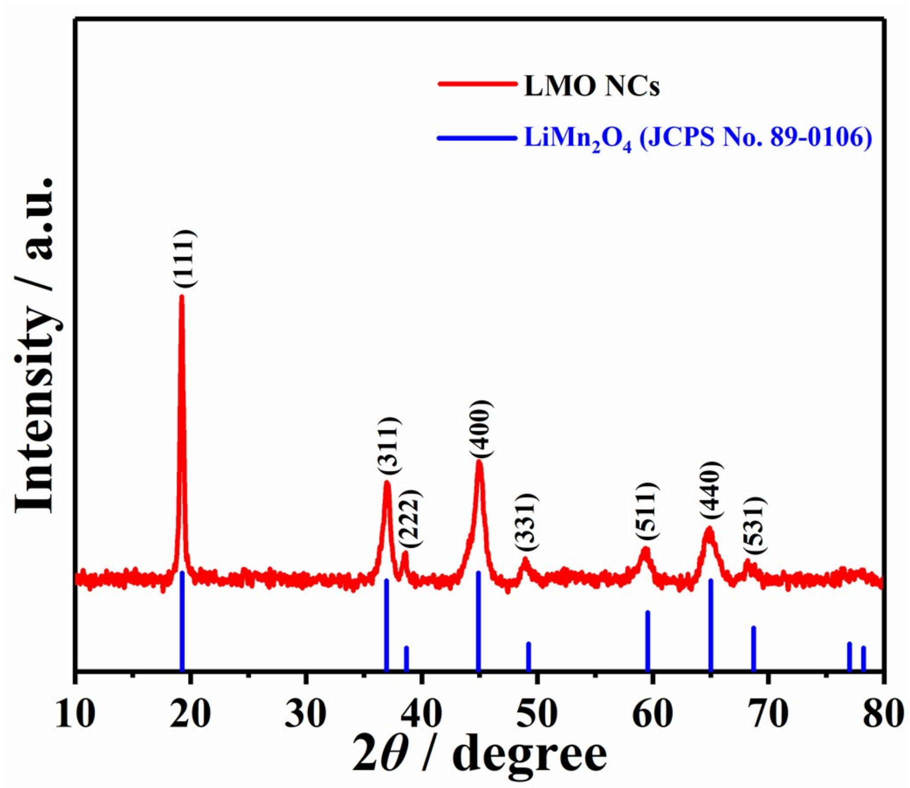

Fig. S6. XRD pattern of LMO NCs.

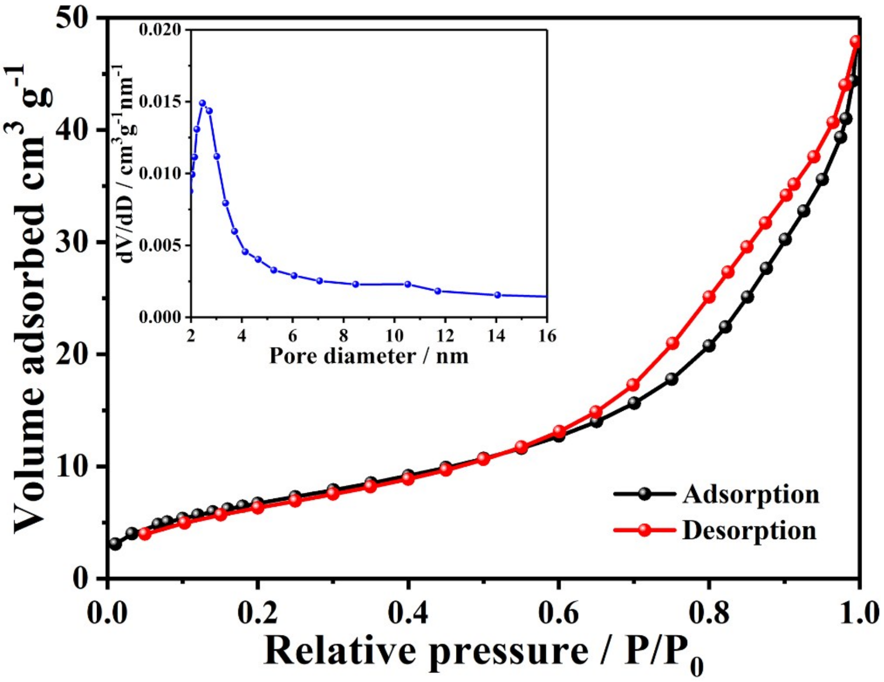

Fig. S7. N_2 adsorption/desorption isotherm curve and the corresponding pore size distributions of $LiMn_2O_4$ nanocrystals.

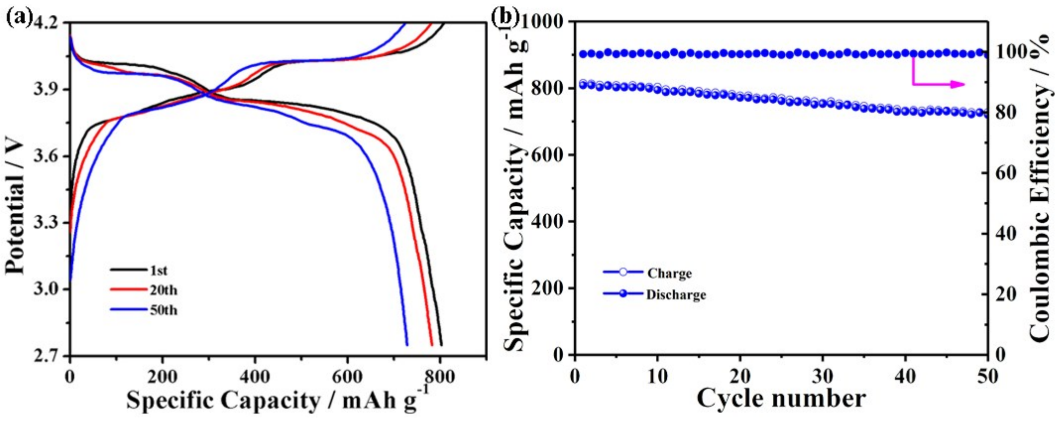

Fig. S8. (a) Galvanostatic charge-discharge profiles and (c) cycling performance of MnO-10/C nanocomposite in full cell, where the specific capacity is calculated according to the weight of MnO-10/C anode.

3. Supplementary Table

Table S1. Comparison of BET specific surface area and the corresponding pore size distribution of MnO-based anode materials.

Samples	BET specific surface area (m^2 g^{-1})	pore size distribution (nm)	References
Porous MnO/C nanotubes	40	7.7	2
MnO@carbon core-shell nanowires	6.86	13	3
MnO/graphite nanosheet	58.1	-	4
MnO@1-D carbon composites	64	24	5
Hierarchical Micro/ Nanostructured MnO	52.5	20/60	6
MnO/C NCs	73.1	3	This work

Table S2. Comparison of electrochemical performance of MnO-based anodes.

Samples	Current density (A g^{-1})	Cycle number	Capacity [a] (mAh g^{-1})	References
Hollow MnO/C microspheres	0.1	50	702.2	7
MnO@C porous microspheres	0.1	100	525.4	8
Porous MnO microspheres	0.05	50	~700	9
Hollow porous MnO/C composites	0.1	50	740	10
MnO thin-films	0.05	100	~700	11
MnO/reduced graphene oxide sheets	0.1	50	665.5	12
MnO/C	0.2	100	~400	13

nanocomposite				
N-doped MnO /graphene	0.1	90	772	14
MnO/carbon microsheets	0.1	90	797.6	15
MnO/C NCs	0.2	150	806.7	This work

[a)] Residual capacity after cycling.

4. Supplementary References

1 J. Park, K. An, Y. Hwang, J. G. Park, H. J. Noh, J. Y. Kim, J. H. Park, N. M. Hwang and T. Hyeon, *Nat. Mater.*, 2004, **3**, 891-895.

2 G. L. Xu, Y. F. Xu, H. Sun, F. Fu, X. M. Zheng, L. Huang, J. T. Li, S. H. Yang and S. G. Sun, *Chem. Commun.*, 2012, **48**, 8502-8504.

3 X. W. Li, S. L. Xiong, J. F. Li, X. Liang, J. Z. Wang, J. Bai and Y. T. Qian, *Chem. Eur. J.*, 2013, **19**, 11310-11319.

4 S. Y. Liu, J. Xie, Y. X. Zheng, G. S. Cao, T. J. Zhu and X. B. Zhao, *Electrochim. Acta*, 2012, **66**, 271-278.

5 X. N. Li, Y. C. Zhu, X. Zhang, J. W. Liang and Y. T. Qian, *RSC Adv.*, 2013, **3**, 10001-10006.

6 G. L. Xu, Y. F. Xu, J. C. Fang, F. Fu, H. Sun, L. Huang, S. H. Yang and S. G. Sun, *ACS Appl. Mater. Interfaces* 2013, **5**, 6316-6323.

7 Y. Xia, Z. Xiao, X. Dou, H. Huang, X. Lu, R. Yan, Y. Gan, W. Zhu, J. Tu, W. Zhang and X. Tao, *ACS Nano*, 2013, **7**, 7083-7092.

8 S. Guo, G. Lu, S. Qiu, J. Liu, X. Wang, C. He, H. Wei, X. Yan and Z. Guo, *Nano Energy*, 2014, **9**, 41-49.

9 K. Zhong, B. Zhang, S. Luo, W. Wen, H. Li, X. Huang and L. Chen, *J. Power Sources*, 2011, **196**, 6802-6808.

10 W. J. Zhu, H. Huang, W. K. Zhang, X. Y. Tao, Y. P. Gan, Y. Xia, H. Yang and X. Z. Guo, *Electrochim. Acta*, 2015, **152**, 286-293.

11 Z. Cui, X. Guo and H. Li, *J. Power Sources*, 2013, **244**, 731-735.

12 Y. J. Mai, D. Zhang, Y. Q. Qiao, C. D. Gu, X. L. Wang and J. P. Tu, *J. Power Sources*, 2012, **216**, 201-207.

13 H. Park, D. H. Yeom, J. Kim and J. K. Lee, *Korean J. Chem. Eng.*, 2015, **32**, 178-183.

14 K. Zhang, P. Han, L. Gu, L. Zhang, Z. Liu, Q. Kong, C. Zhang, S. Dong, Z. Zhang, J. Yao, H. Xu, G. Cui and L. Chen, *ACS Appl. Mater. Interfaces*, 2012, **4**, 658-664.

15 J. L. Liu, N. Chen and Q. M. Pan, *J. Power Sources*, 2015, **299**, 265-272.
